# Dynamic Tuning of a Thin Film Electrocatalyst by Tensile Strain

**DOI:** 10.1038/s41598-019-52245-y

**Published:** 2019-11-04

**Authors:** Eric E. Benson, Mai-Anh Ha, Brian. A. Gregg, Jao van de Lagemaat, Nathan R. Neale, Drazenka Svedruzic

**Affiliations:** National Renewable Energy Laboratory, Golden, CO 80401 USA

**Keywords:** Chemistry, Energy science and technology, Materials science

## Abstract

We report the ability to tune the catalytic activities for the hydrogen evolution reaction (HER) and oxygen evolution reaction (OER) by applying mechanical stress on a highly n-type doped rutile TiO_2_ films. We demonstrate through *operando* electrochemical experiments that the low HER activity of TiO_2_ can reversibly approach those of the state-of-the-art non-precious metal catalysts when the TiO_2_ is under tensile strain. At 3% tensile strain, the HER overpotential required to generate a current density of 1 mA/cm^2^ shifts anodically by 260 mV to give an onset potential of 125 mV, representing a drastic reduction in the kinetic overpotential. A similar albeit smaller cathodic shift in the OER overpotential is observed when tensile strain is applied to TiO_2_. Results suggest that significant improvements in HER and OER activities with tensile strain are due to an increase in concentration of surface active sites and a decrease in kinetic and thermodynamics barriers along the reaction pathway(s). Our results highlight that strain applied to TiO_2_ by precisely controlled and incrementally increasing (i.e. *dynamic*) tensile stress is an effective tool for dynamically tuning the electrocatalytic properties of HER and OER electrocatalysts relative to their activities under static conditions.

## Introduction

The ability to alter a materials’ structure/function relationship by strain has been widely recognized^1^. Electrocatalytic activities of heterogenous catalysts depend on the surface reactivities toward chemical species along a reaction pathway. Surface reactivities are highly dependent on the surface electronic state, crystal structure and concentration of accessible active sites, all of which can be modulated by surface strain. Several recent reviews summarize theoretical and experimental studies how strain affects electrocatalytic materials^2–4^. Surface strain can be introduced either internally through material architecture (*static strain*) or by applying an external force (*dynamic strain*). Static strain in crystalline materials can be introduced by doping^5^, de-alloying^6,7^, annealing^8^, epitaxial growth on a mismatched crystal lattice^2,3,9,10^ or by intrinsic surface-stress in 2D materials^11^. In polycrystalline materials, strain naturally occurs within grain boundaries due to crystals twinning or edge defects. Due to the size confinement, nanomaterials are inherently strained, and that strain can be tuned by preparing nanoparticles with various shapes or sizes^2,3^. For architecturally strained materials, it is often difficult to separate strain-induced effects from chemical or ligand effects^12–14^.

Tuning static strain by varying material architecture is a rather laborious approach since it requires synthesis of a new sample for each discreet amount of strain. The ability to systematically measure materials’ structure/function relationship under precisely controlled and incrementally increasing strain allows one to explore a dynamic range over the strain space without introducing other effects. Experimental studies showing the effects of dynamic strain on electrocatalytic activities have emerged in the literature only recently. A comprehensive review of strained electrochemical systems was published recently^15^. Examples of tunable substrates include elastic materials such as organic polymers^16–21^ and metallic materials such as stainless steel^22^ and pseudoelastic/shape-memory NiTi alloys^23,24^. Alternatively, external forces have been applied by an atomic force microscopy tip^25^, by introducing subsurface inert gas bubbles^26–28^ or by Li-ion intercalation/deintercalation in battery materials^6,29–31^. Application of mechanical, thermal or electrical loading result in bending, compression or expansion of an elastic substrate, further inducing stress-strain response on the deposited material. Such catalyst engineering through dynamic strain has been shown for: *i*) HER on MoS_2_, Au, Pt, Ni, Cu, WC^16–19^ and *ii*) OER on NiO_x_^22^, nickel-iron alloys^32^. The primarily focus of the previous studies was effects of strain on the catalytic activities of transition metals and the experimental results were consistent with d-band theory, described in the seminal work by Mavrikakis *et al*.^33^. In contrast, effects of strain on catalytic properties of materials with more complex chemical and electronic structures, such as metal oxides, remains poorly understood. In addition to d-band, strain can affect overlap of d and p orbitals from metal and oxygen atoms, respectively, thereby inducing M–O bond rearrangement and phase transitions^34,35^. Strain can also affect formation energies and diffusion pathways of oxygen vacancies (V_O_), leading to changes in surface reactivities^26,36–42^. While TiO_2_ has been shown to split water under illumination, a co-catalyst (typically Pt) is used to promote catalysis. Although the stochiometric rutile TiO_2_ surface has low reactivity toward water, theoretical studies suggested that the surface reactivity can be activated by tensile strain^37,43^. Recently, scanning tunneling microscopy (STM) measurements showed increase in hydrogen (H*) adsorption energy on stochiometric rutile TiO_2_(110) with increase in surface strain^26,27^. Due to the complexity of the system, most of the previous studies were focused on theory and only specific aspects of TiO_2_ reactivity with water as a function of strain. Considering that strain can affect simultaneously multiple aspects of TiO_2_ electronic structure and reactivity, it can be expected that each step along a reaction pathway is affected to some degree by strain. Here we show and discuss the effects of dynamic tensile strain on TiO_2_ HER and OER activities, based on both experiments and theory. The effects, reported here, are significantly larger than ones observed for transition metals catalysts and trend is reversed.

## Results and Discussion

**Figure 1:**
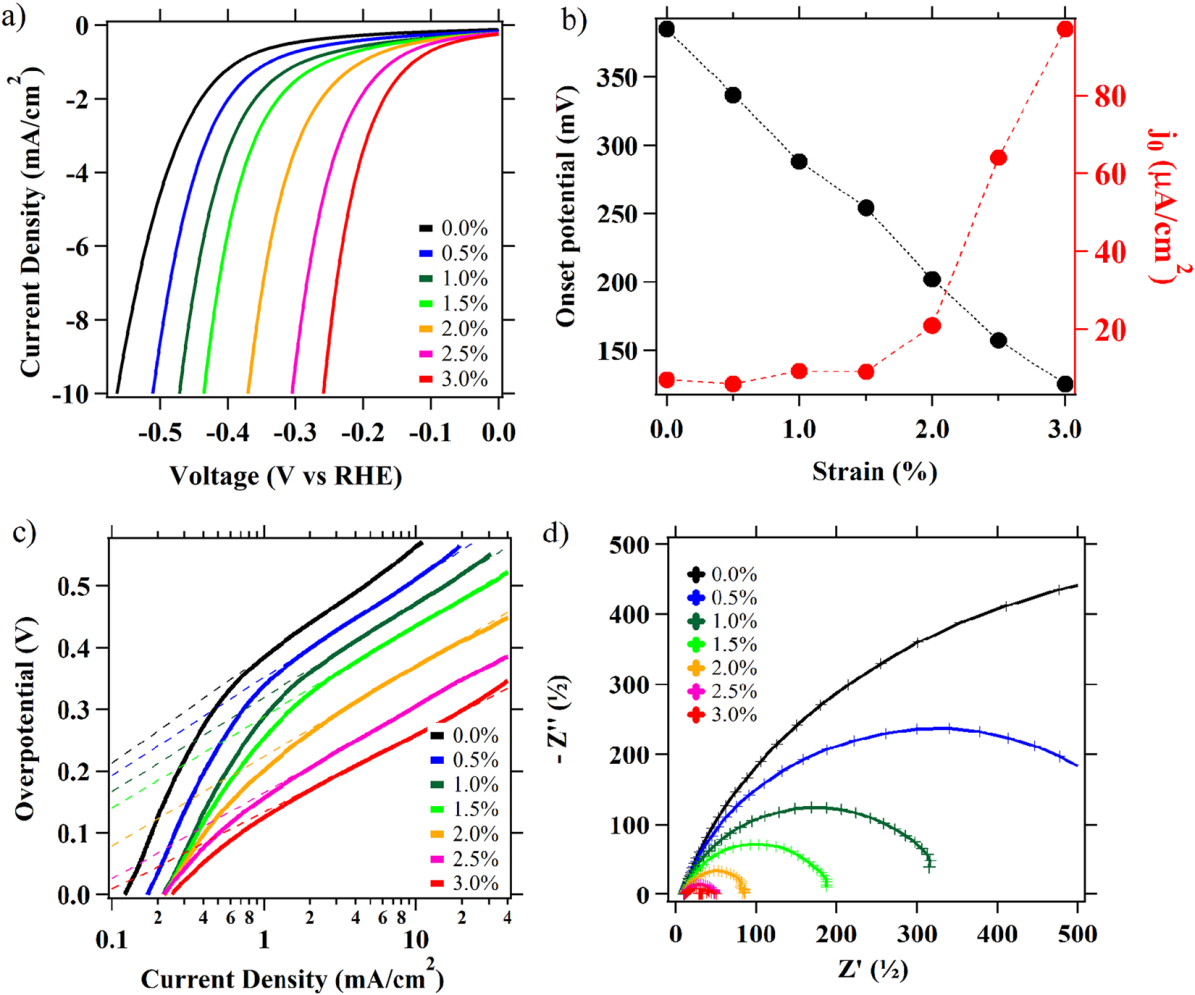
(**a**) Linear sweep voltammetry (LSV) experiments with TiO_2_ films in 0.5 M sulfuric acid, at scan rates 50 mV/s. (**b)** Overpotentials for 1 mA/cm^2^ current densities and exchanged current densities determined from Tafel plots are given for comparison. (**c)** Tafel plots. (**d)** Electrochemical impedance at −0.38 V vs. RHE from 1 Hz to 100 kHz (Nyquist plots).

The experimental set-up used in this study was described in our preceding publication^39^. Briefly, rutile TiO_2_ thin films are thermally grown on a pseudo-elastic material Nitinol (NiTi intermetallic). Due to the oxophilic nature of titanium, thermal treatment of NiTi at elevated temperatures under aerobic conditions leads to a nickel-free surface of TiO_2_. Oxidation of mechanically polished NiTi at 500 °C for 30 min results in a ∼50 nm thick films of rutile TiO_2_(110), confirmed by XPS, XRD and Raman spectroscopies^39^. In this work, we find that electrocatalytic results are the most reproducible and effects of strain highest for samples that are never stressed past 3%. For detailed experimental protocols describing sample preparation, application of tensile strain and electrochemical experiments see the Supplemental Information section. The strain applied to the thermally treated, TiO_2_-coated NiTi foil is increased at 0.5% increments from 0 to 3%, (% corresponds to an increase in electrode surface from its original dimensions). HER catalytic activities were evaluated by steady-state electrochemistry measurements in 0.5 M sulfuric acid aqueous solution with TiO_2_ films under dynamic tensile strain a 0–3% (Fig. S1a,b). Linear sweep voltammetry (LSV) results are shown in Fig. 1a. A summary of the electrochemical parameters can be found in Tables 1 and S1. As the samples are strained, the overpotential (η, taken as the voltage required to pass 10 mA/cm^2^) shifts anodically by a remarkable ∼320 mV (Fig. 1b). The TiO_2_ under zero applied strain shows a large Tafel slope (173 mV/dec) at overpotentials where Tafel behavior is observed and a small exchange current density (j_0_ = 7.0 μA/cm^2^) indicative of a poor HER catalyst (Fig. 1c). At 3% strain, the Tafel slope decreases to 124 mV/dec at overpotentials above 100 mV and the exchange current density increases 14-fold to 97 μA/cm^2^. The Tafel slope of around 120 mV/dec suggests that the rate limiting steps under those conditions are defined by the Volmer or Volmer-Heyrovsky reaction mechanism (Eqs 1, 2; * denotes a surface site)^44^.

1 $${\rm{V}}{\rm{o}}{\rm{l}}{\rm{m}}{\rm{e}}{\rm{r}}\,{\rm{s}}{\rm{t}}{\rm{e}}{\rm{p}}:\,{{\rm{H}}}_{3}{{\rm{O}}}^{+}+{{\rm{e}}}^{-}+\ast \leftrightarrows {\rm{H}}\ast +{{\rm{H}}}_{2}{\rm{O}}$$

2 $${\rm{H}}{\rm{e}}{\rm{y}}{\rm{r}}{\rm{o}}{\rm{v}}{\rm{s}}{\rm{k}}{\rm{y}}\,{\rm{s}}{\rm{t}}{\rm{e}}{\rm{p}}:\,{\rm{H}}\ast +{{\rm{H}}}^{+}+{{\rm{e}}}^{-}\rightleftarrows {{\rm{H}}}_{2}+\ast $$

**Table 1:** HER electrochemical parameters measured for TiO_2_ electrodes at 0–3% strain. More information is given in Table S1. HER activities are measured in 0.5 M H_2_SO_4_. EIS measurements are conducted in the frequency range 1 Hz to 100 kHz at −0.38 V vs RHE.

Strain (%)	*-η* (mV) at 1 mA/cm^2^	*-η* (mV) at 10 mA/cm^2^	j_0_ (A/cm^2^)	Tafel slope (mV/dec)	R_CT_ (Ω)	C_dl_ (μF)
0	385	565	7 × 10^−6^	173	1108	4.1
1	289	472	9 × 10^−6^	155	332	3.8
2	202	371	21 × 10^−6^	137	88	5.1
3	125	260	97 × 10^−6^	124	39	7
Literature^47–49^	—	30-340	10^−2^–10^−9^	30–50	—	—

The higher Tafel slopes at low overpotentials are often observed for semiconductors, where charge transfer is mediated by surface states^45,46^. We also conduct electrochemical impedance spectroscopy (EIS) measurements in the frequency range 1 Hz to 100 kHz at different strain conditions. Nyquist plots (Fig. 1d) show that the high frequency series resistance (R_S_) (∼10 Ω) which is normally mostly determined by conduction in the electrolyte does not change significantly with strain, suggesting that strain has no significant effects on the reaction conditions. In contrast, the charge transfer resistance (R_CT_) decreases systematically from ∼1.1kΩ to ∼40 Ω for 0 to 3% strain, respectively. The decrease in R_CT_ represents an improvement in the reaction kinetics between TiO_2_ surface and reactants in solution. As can be expected, the observed decreases in R_CT_ with strain are inversely proportional to the observed increases in j_0_. Overall, the measured electrochemical parameters suggest that improved HER activities under strain are due to decreased overpotential (*η*), increased concentration of active surface sites (i.e. higher j_0_) and consequently faster reaction kinetics (i.e. higher *η*, j_0,_ 1/R_CT_). Interestingly, j_0_ and 1/R_CT_ do not linearly vary with strain and more significant changes in electrochemical parameters (η, j_0,_ R_CT_) occur for strains above ∼1.5% (Fig. 1b, S2a–c). Electrochemical data are consistent with the results we reported previously, where strain raises the energy distribution of V_O_s (n-type dopants) near the conduction band and causes an increase in carrier concentration density of surface states (SS_DOS_), with most significant increase between 1–2% (Fig. S2d)^39^.

In sum, these electrochemical experiments show that straining a rutile TiO_2_ film transforms it from a poor HER electrocatalyst to a facile one, with activities comparable to activities of other state-of-the-art earth-abundant metal catalysts^47–49^. For example, the HER onset potential at 3% strain is comparable to these typically reported for molybdenum (MoS_2_) and tungsten sulfides (WS_2_) (∼250 mV at 10 mV/cm^2^)^49^, while exchange current densities (∼10^–4^ A/cm^2^) are significantly higher^48,49^.

**Figure 2:**
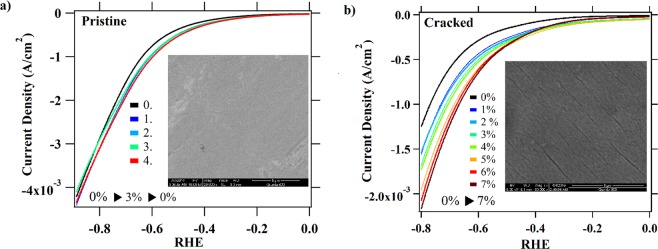
(**a**) LSV measured with sample that underwent 4 strain-release cycles between 0 and 3%. After each cycle the LSV is measured at 0% strain. Insert: SEM images of sample strained to 3%. (**b)** LSV data for the cracked sample collected for strain incrementally increased 0 to 7%. Insert: SEM image of sample kept at 7% strain overnight, after which the sample is released. Obvious surface fissures are visible. All LSV data are collected in 0.5 M H_2_SO_4_.

Generally, the effects of mechanical strain on any solid material can lead to changes in grain reorganization and/or changes in crystal structure at atomic level. Effects, such as cracking and fissuring of the TiO_2_ film could in theory improve HER activities by *i*) exposing more catalytically active TiO_2_ crystal facets, edges or defects, *ii*) increasing the overall electroactive surfaces or *iii*) exposing the underlying NiTi substrate. We have explored each of these conceivable circumstances in more detail. First, we note that our prior work on thermally grown rutile TiO_2_ on Nitinol foils showed elastic behavior, with no cracking at low tensile strain values of 0–5%^39^. Here we confirm those results by imaging surface morphology with strain using scanning electron microscopy (SEM). SEM images were taken of polished samples, after oxidation at 500 °C, first strained multiple times to 3% (i.e. elastic range) and then to 7% (i.e. inelastic range). When strained to 3% no change in the TiO_2_ surface morphology is observed (Fig. 2a, insert). However, when samples are strained up to 7% a change in the surface morphology (fissuring) is observed and large cracks in the surface are visible (Fig. 2b, insert). SEM findings are consistent with the electrochemical data. There are no significant changes in HER activities at 0% strain for samples that undergo multiple stretch-release cycles up to 3% and the effects of strain on HER activities were reversible (Fig. 2a). After the first strain-release cycle, small permanent increases in HER activity is observed, likely due to some surface activation process. For samples that are purposely cracked by straining past their elastic limit, some increases in HER activities with strain are observed (Fig. 2b). Nevertheless, increase in HER are less significant than ones presented in Fig. 1a and the effects are irreversible.

Additional evidence that the overall electroactive surface is not increasing significantly with strain is provided by electrochemical analysis. Double layer capacitance (C_dl_) measurements show that the C_dl_ increase only 1.7-fold from 4.1 to 7.0 μF when strained from 0 to 3% (Table 1, Fig. S2b), which is significantly lower than changes in observed HER activities. LSV measurements also are conducted with NiTi substrates that were not thermally treated (*i*.*e*., simply the native oxide NiTiO_x_ without a thermally grown rutile TiO_2_ overlayer). For this control we observe a small, reversible increase in HER activities with strain, with about ∼10 mV cathodic shift in HER onset potential per 1% percentage strain (Fig. S4). Although relatively small, the effects of strain on HER activities for untreated NiTi are still significant and comparable to previous reports on strain-induced changes in HER activities for metallic substrates (5–30 mV/% strain^17,18^). For comparison, we see about ∼100 mV change in onset potential per 1% strain for thermally grown 50 nm thick TiO_2_ film. Other electrochemical parameters for untreated NiTiO_x_ also are inconsistent with those measured for TiO_2_ (Table S1). These data make clear that exposure of the NiTiO_x_ underlayer via cracking cannot explain increases in TiO_2_ HER activities. In total, these results strongly suggest that *i)* opening and closing of surface fissures and exposure of the NiTiO_x_ substrate does not explain large improvements in HER activities we have observed; and *ii*) a continuous TiO_2_ film is required to observe large, reversible effects of strain suggesting an elastic deformation.

**Figure 3:**
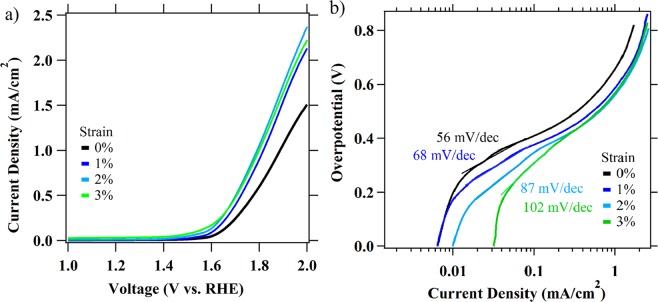
OER activities measured in 1 M NaOH aqueous solution at scan rates 50 mV/s. (**a**) Linear sweep voltammetry experiments with TiO_2_ films under tensile strain (0–3%) and (**b**) Tafel plots.

In addition to HER, we also examine the effects of tensile strain (0–3%) on the rutile TiO_2_ activity for the OER. LSV measurements in 1 M NaOH are conducted with 50 nm thick TiO_2_ rutile thermally grown on NiTi (Fig. 3a). The LSV curves show large onset potentials (η, defined as the potential at an OER current density of 1 mA/cm^2^) (Table 2) in comparison to other OER catalysts^48,49^. The η required to pass 1 mA/cm^2^ shifts cathodically 89 mV from 0 to 3% strain. In comparison to HER activities presented in this work, the observed increases in OER activities with strain are more moderate, but nevertheless comparable to the previous studies on strain-induced effects on OER for different materials^6,22,32,38^. Tafel analysis shows increase in Tafel slope with 0–3% strain suggesting strain effects OER mechanism (Table 2, Fig. 3b). Significant increases in exchange current densities (j_0_) with increased strain (Table 2, Fig. 3b) are consistent with increased concentration of surface active sites and faster reaction kinetics. Similar to HER results, OER data are consistent with our previous study, where strain raises the energy distribution of V_O_s (n-type dopants) near the conduction band and causes an increase in carrier concentration density of surface states (SS_DOS_)^39^. Interestingly, Liu *et al*. observed an opposite trend with pervoskite cobaltite thin films, where OER activities decrease under applied static tensile strain or with introduction of oxygen vacancies^50^. Further increases in OER activities above 3% strain (Fig. S6) are due to some TiO_2_ film fissuring. This can be expected considering that surface fissuring exposes Ni, which in its oxidized form (NiO_x_) is a better OER catalyst than TiO_2_ in the alkaline electrolyte investigated here. As with the HER results, for cracked TiO_2_ surface effects of strain on OER activities are irreversible.

**Table 2:** OER electrochemical parameters measured for TiO_2_ electrodes at 0–3% strain. OER activities are measured in 1 M NaOH.

Strain (%)	*-η* (mV) at 1 mA/cm^2^	j_0_ (A/cm^2^)	Tafel slope (mV/dec)
0	1.89	0.5 × 10^−9^	59
1	1.82	1.7 × 10^−8^	68
2	1.79	6.6 × 10^−7^	87
3	1.80	4.5 × 10^−6^	102

### Theory

In our preceding study with n-doped TiO_2_ films we showed that the tensile strain applied on 50 nm rutile TiO_2_ primarily affects the spatial and energetic distribution of oxygen vacancies (V_O_s)^39^. Therefore strain-induced changes in the HER and OER catalytic activities are associated with the changes in V_O_s. This is consistent with previous reports showing that bridging V_O_s are primary active sites for dissociative water adsorption^51–54^. Water dissociation is followed by proton transfer to nearby bridging oxygen atom (OH_b_) forming two hydroxyl groups for each V_O_, and finally diffusion of OH_b_ away from the original binding site^53^. In contrast to defective TiO_2_, the surface of stochiometric TiO_2_ (s-TiO_2_) is considered unreactive toward water molecules. However, tensile strain can increase water reactivity on s-TiO_2_ by increasing the energy gain upon water adsorption and by decreasing its dissociation barrier^37^.

**Figure 4:**
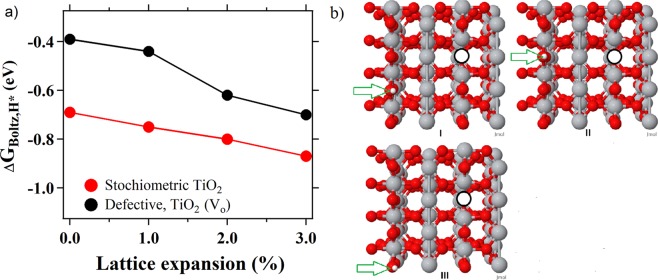
(**a**) Boltzmann-weighted free energy (∆G_Boltz,H*_) versus the strain of lattice expansion with the results on the stoichiometric surface in black and the defective surface with an oxygen vacancy (V_O_) in grey. (**b)** Local minima of H* on the defective TiO_2_ (110) surface. The same minima were found on the strained surfaces, but in a different order with corresponding changes to the relative energies as detailed Table S6. V_O_ is denoted by an outlined circle, Ti atoms are gray, oxygen atoms are red, green arrow is added for an easier indentification of H*.

For most transition metal oxides, the first step in HER mechanism (Eq. 1) is considered a facile chemical reaction. Typically, the common descriptor for HER activities is hydrogen adsorption free energy (ΔG_H*_), with the most efficient HER catalysts having ΔG_H*_ approaching 0 (*i*.*e*., Sabatier principle). Tensile strain can increase or decrease H* binding in a manner that depends on a catalyst electronic state (*d-band theory* for transition metals)^33,55^, as well as an applied overpotential^16^. A previous study with cobalt(II) oxide nanorods, where strain was imposed through nanostructuring, showed that an increase in tensile strain from 0 to 4% leads to an increase in ΔG_H*_ from negative to positive values, with optimal ΔG_H*_ around 0 eV achieved at 3% strain^40^. To study H* adsorption for our system, we performed Plane-Wave Density Functional Theory (PW-DFT) calculations for strained rutile TiO_2_ (110) surfaces (both stochiometric and defective TiO_2_). Detailed description of the computational methods is given in the Supplemental Information section. Calculations show that the adsorption strength of H* is weakened in the presence of an V_O_ (Table S3,S5) with the local minima of H* configurations choosing bridging oxygen sites in the row opposite of the V_O_ (see Fig. 4b for the three most stable H* configurations). Boltzmann-weighted adsorption free energies plotted against 0–3% strain are summarized in Fig. 4a and Table S5. Depending on the strain applied, the lowest three configurations change in stability with concomitant changes in the Boltzmann populations; we note that all three configurations are often nearly degenerate (<0.5 eV difference in energy) and highly accessible (Fig. 4b, Table S6). In other words, the energetic differences in binding of H* to O_b_ in the row opposite OV is considerably more stable than a site near the V_O_ (configuration IV in Supplemental Information Fig. S9, Table S6 is less stable by >0.3 eV). In sum, we found that tensile strain imposed on TiO_2_ increases both H* binding (i.e. more negative ΔG_H*_) and HER activities measured experimentally. This trend is opposite from the one observed for CoO nanorods described above, as well as the trend for transition metal catalysts where excessive H* binding impedes HER activities^17–19^. Tafel slopes between 173 and 120 mV/dec measured in this work (Table 1) are higher than ones reported for CoO nanorods^40^ as well as most of other transition metal catalysts^18,49^. Higher Tafel slopes suggest different HER mechanism, possibly one with more significant impact of the first Volmer step (Eq. 1). Hypothetically, more negative ΔG_H*_ can improve HER activities by decreasing activation barrier or by improving thermodynamics for H* formation (Eq. 1, Fig. S10). Alternatively, overall HER rates could be limited by a modest reactivity of water molecules on TiO_2_ surface, and not H* binding. Both hypotheses are consistent with higher Tafel slopes determined experimentally and presented computational results. More comprehensive understanding how strain affects HER and OER catalytic mechanisms requires additional computation studies for the entire reaction profile, especially looking at interaction of water molecules with V_O_s. Such calculations are rather complex and would exceed the scope of this publication.

It is important to recognize that effects of strain on TiO_2_ HER activities are very complex. Relatively simple theoretical model with single V_O_ and single H* is presented here. Previous reports show that applied strain effects diffusion pathways and overall distribution of H*s on rutile TiO_2_^53,56^. Strain also affects formation, diffusion and energy of V_O_s, as discussed throughout the text. Our calculations show that the formation energy required to create an V_O_ decreases with increasing tensile strain from 2.86 eV (unstrained) to 2.51 eV (strained at 3%) (Table S2). Therefore, it is likely that strain influences the mechanism of water splitting leading to H_2_ evolution in more complex ways than simply changing the adsorption strength of H*. Effects of strain on V_O_s will lead to considerable effects on reaction barriers and pathways to water splitting, where the optimum pathway might differ depending on strain.

## Conclusions

We showed that dynamically straining a thin film of n-doped rutile TiO_2_ up to 3% tensile strain using an elastic NiTi substrate significantly increases both HER and OER activities. Significant improvements in HER activities with tensile strain are likely due to an increase in surface active sites and a decrease in kinetic and thermodynamics barriers along the reaction pathway(s). In our preceding work^39^ we showed that tensile strain increased density of surface accessible V_O_s, which is consistent with improved HER and OER activities. We calculate a lower activation barrier for V_O_ formation and a stronger binding of the H* intermediates with strain. This study demonstrates that application of mechanical stress may be a general method for tuning dynamically the catalytic properties of metal oxides.

## Methods

In this work we followed the procedures we published earlier^39^. Briefly, superelastic NiTi foil (0.05 mm thickness) was obtained from Alpha Aesar and cut into ~1 × 5 cm samples. The foils were then oxidized at 500 °C under aerobic conditions for 30 minutes. Oxidized NiTi samples were loaded into an *MTI/Fullam SEMTester* equipped with a 450 N capacity load cell and controlled using *MTESTQuattro* control software. Samples were strained at a rate of 2 mm/min. Electrochemical measurements were controlled by a *CH Instruments 600D* potentiostat using a custom-built single compartment cell with an Ag/AgCl reference electrode and platinum counter (Fig. S1a), we have described in our previous publication^39^. For a typical experiment, the cell is loosely assembled around the NiTi sample and then the sample is pre-strained to 5 N, the cell is then tightened onto the sample to create a solution tight cell for electrochemical measurements. To strain the working NiTi electrode, the electrolyte is drained and the cell loosened so that the sample can move freely, and then the strain is adjusted (2 mm/min) under software control. The cell is then re-aligned, gently tightened back onto the sample, and the electrolyte is replaced for further measurements. This procedure is repeated at each strain value (Fig. S1b). We observed that electrochemical results were the most reproducible and effects highest for samples that was never stretched pass 3%. To make sure that observed increases in hydrogen evolution reaction (HER) and oxygen evolution reaction (OER) were not due to the electro-deposition of trace amount of platinum on the working electrode from counter Pt electrode, we run control with carbon felt electrode. Silver/silver chloride and mercurous oxide were used as a reference electrode for HER or OER measurements respectively.

## Supplementary information

Supplementary information
