# Supplementary material for: Dynamic Tuning of a Thin Film Electrocatalyst by Tensile Strain
Source: Sci Rep. 2019 Nov 4;9:15906. doi: 10.1038/s41598-019-52245-y (PMC6828675; doi:10.1038/s41598-019-52245-y)
Supplement: Supplementary file 1 — Supplementary information [file 41598_2019_52245_MOESM1_ESM.docx]

*Supplementary Information for:*

**Dynamic Tuning of a Thin Film Electrocatalyst by Tensile Strain**

Eric E. Benson, Mai-Anh Ha, Brian. A. Gregg, Jao VandeLagemaat, Nathan R. Neale, Drazenka Svedruzic*

National Renewable Energy Laboratory, Golden, CO 80401, USA. E-mail: Drazenka.Svedruzic@nrel.gov

**METHODS**

**b.**

**Nitinol (NiTi) Preparation.** Superelastic NiTi foil (0.05 mm thickness) was obtained from Alpha Aesar and cut into ~1 x 5 cm samples. Each sample was then mechanically polished using 320, 600, and 1200 grit sandpaper. After polishing, the samples were sonicated for 5 min in sequential baths of MQ H_2_O, isopropanol, MQ H_2_O and then dried under nitrogen. The foils were then oxidized at 500 ⁰C under aerobic conditions for 30 minutes.

**a.**


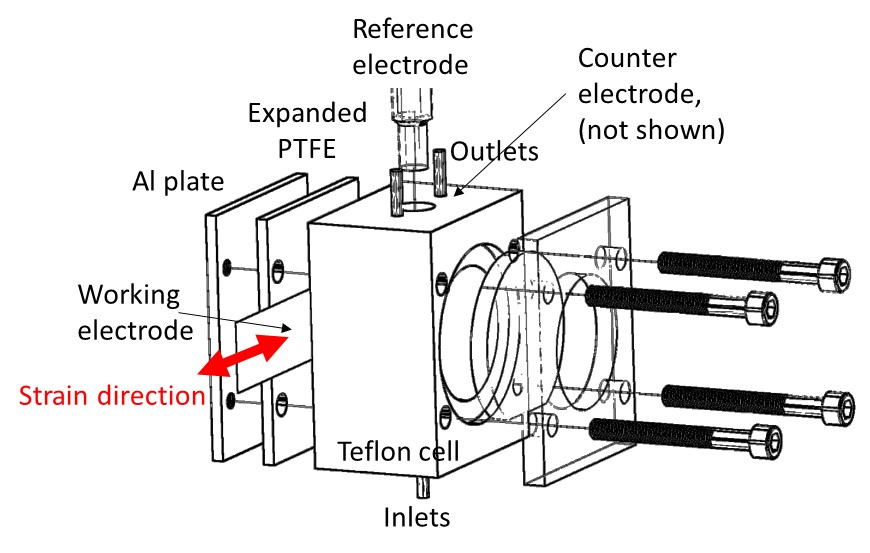


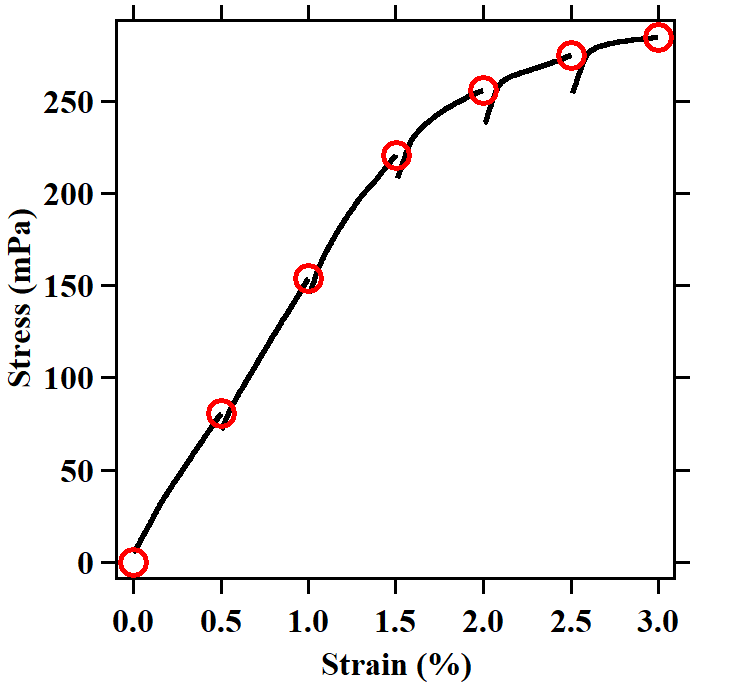


**Figure S1.** **a.** Schematic of the electrochemical cell used for HER and OER measurements under tensile strain. **b.** Stress-strain curve measured for Nitinol foil (1 cm x 5 cm) with deposited TiO_2_ (50nm) using MTI/Fullam SEMTester. Stress can be defined as force applied per unit area, while strain can be defined as a material deformation due to the applied stress (expansion in this case). Red circles represent points where electrochemical measurements are conducted.

**RESULTS**

**Hydrogen Evolution Activities Under Tensile Strain**

**Table S1**. Electrochemical parameters for HER measured for 50 nm rutile TiO_2_ film under 0-3 % tensile strain in 0.5 M sulfuric acid (Figure 1, S1). Onset potential (η) were defined as potential at which 1 or 10 mA/cm^2^ current densities were achieved, which was chosen for easier comparison with literature data.

| **Strain (%)** | ***-η* at 1 mAcm^-2^ (mV)** | ***-η* at 10 mAcm^-2^ (mV)** | **j_0_**  **(μAcm^-2^)** | **Tafel slope (mVdec^-1^)** | **Rs**  **(Ω)** | **R_CT_**  **(Ω)** | **C_dl_**  **(μF)** |
| --- | --- | --- | --- | --- | --- | --- | --- |
| *50 nM TiO_2_ rutile (thermally treated NiTi)* | | | | | | | |
| 0 | 385 | 564 | 7.0x10^-6^ | 178 | 9.81 | 1108 | 4.1 |
| 0.5 | 338 | 511 | 5.9 x10^-6^ | 158 | 9.96 | 614 | 3.7 |
| 1 | 289 | 472 | 9.2 x10^-6^ | 155 | 10.1 | 332 | 3.8 |
| 1.5 | 255 | 436 | 9.1 x10^-6^ | 143 | 10.4 | 187 | 4.3 |
| 2 | 202 | 371 | 2.1 x10^-5^ | 137 | 10.6 | 88.2 | 5.1 |
| 2.5 | 158 | 306 | 6.4 x10^-5^ | 139 | 10.8 | 40.8 | 5.9 |
| 3 | 126 | 260 | 9.7 x10^-5^ | 124 | 11.2 | 39.2 | 7.0 |
| Change 0-3% | -259 mV | -304 mV | 14-fold | -54 mVdec^-1^ | 1.1-fold | 0.03-fold | 1.7-fold |
| *Polished NiTi (not thermally treated, NiTiO_x_)* | | | | | | | |
| 0 | 248 | 422 | 3.8 x10^-5^ | 176 | - | - | - |
| 3 | 214 | 401 | 7.5 x10^-5^ | 181 | - | - | - |
| Change 0-3% | -34 mV | -19 mV | 1.9-fold | 5 mVdec^-1^ | - | - | - |
| *Pt foil (unstrained)* | | | | | | | |
| 0 | 14 | - | 3.82 x10^-4^ | 41 | - | - | - |
| *Values reported in literature for various catalyst for HER in acetic pH*[*^1^*](#_ENREF_1)*^,^*[*^2^*](#_ENREF_2) | | | | | | | |
| 0 |  | 30-340 | 10^-2^-10^-9^ | 30-50 | - | - | - |

***a) Electrochemical parameters measured for TiO2***

**a.**

**b..**

**d..**


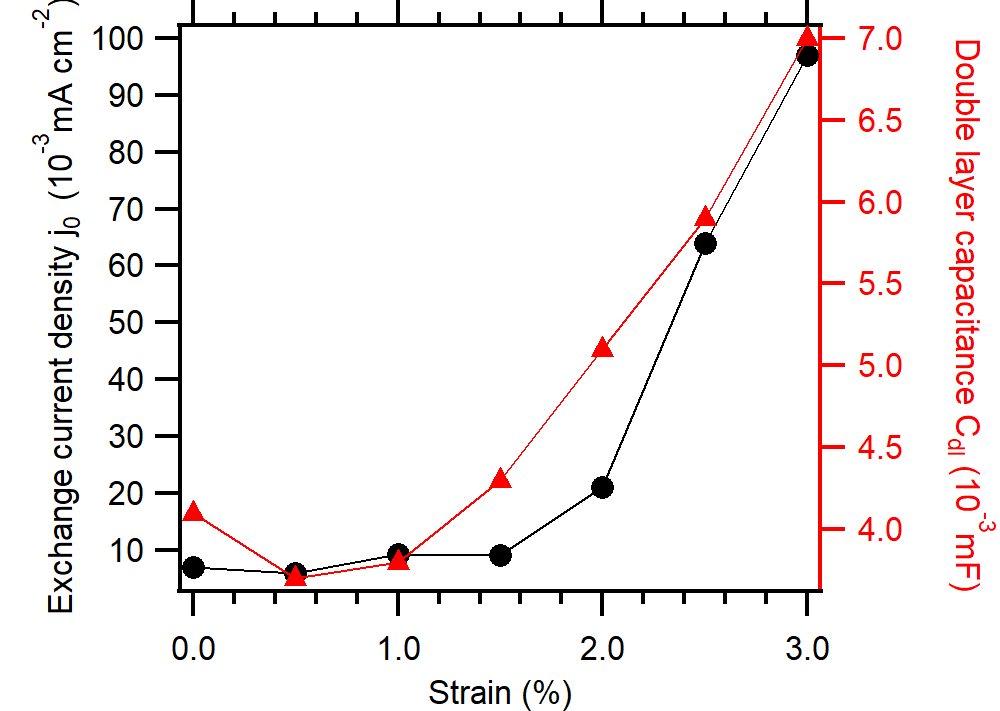

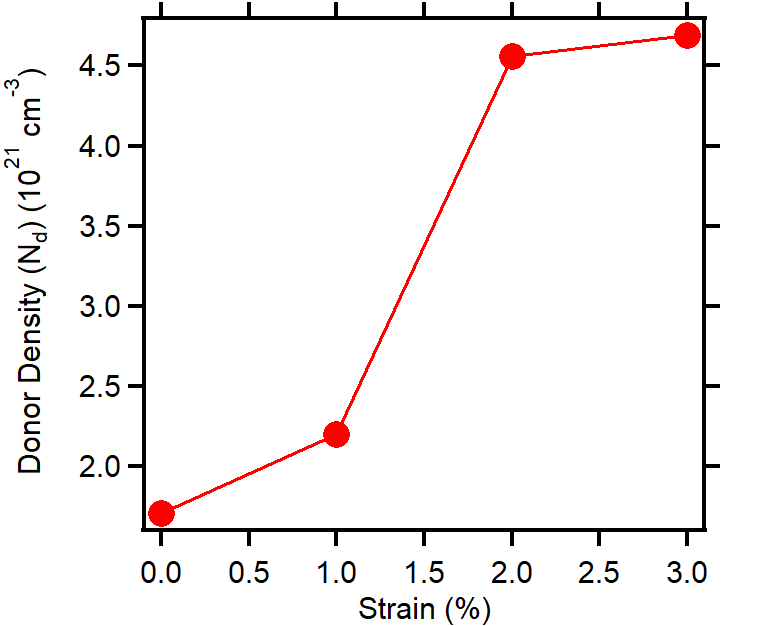


**c..**


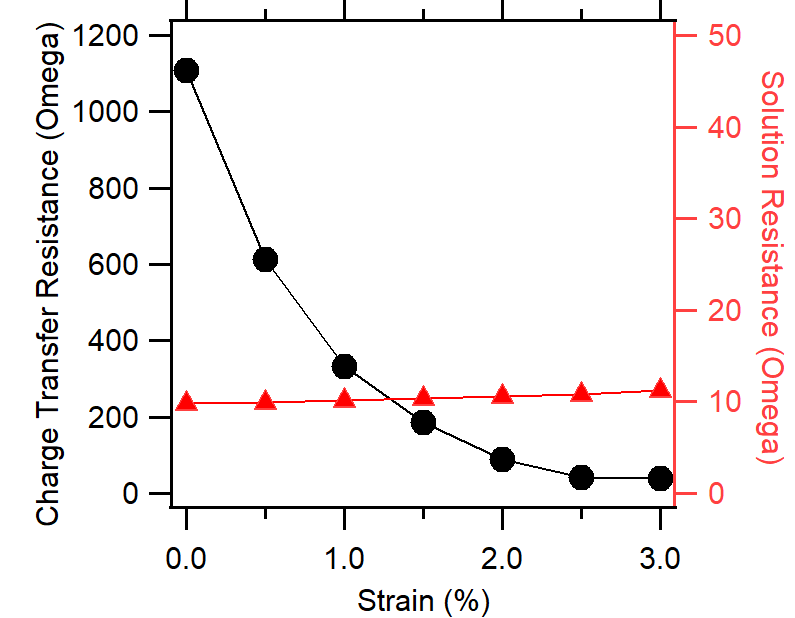


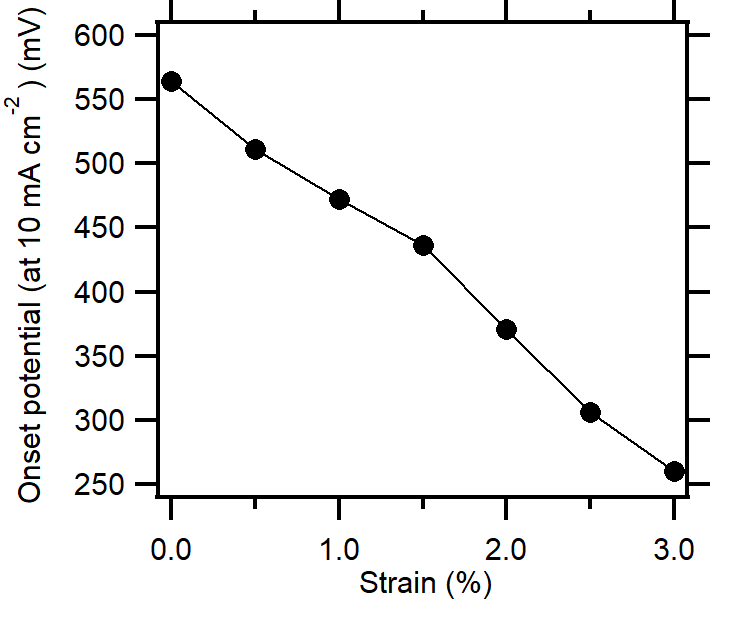


**c.**

**Figure S2.** Summary of measured electrochemical parameters for different strain conditions: **a.** onset potential (η), **b.** exchange current density determined from fitting Tafel plots (Figure 1c), double layer capacitance (from Figure S3), **c.** charge transfer and solution resistance from impedance data (Figure 1d) **d**. Donor densities determined in proceeding work of the same system[^3^](#_ENREF_3). Lines between data points are added to guide eye. Note, for all parameters (other than Rs) the changes with strain are not linear, with the most significant changes for strain a ~1%.

**Table S2.** ESI data from Figure 1d. To obtain R_S_ and R_CT_ data were fitted to (x-a)^2^+(y-b)^2^=r^2^ function.

| Data Series | Fraction data used | Fit center x | Fit center y | Fit left x intercept | Fit Radius |
| --- | --- | --- | --- | --- | --- |
| 1 | 0.85 | 545 | -143.5 | 9.81 | 554 |
| 2 | 0.85 | 308 | -73.4 | 9.96 | 307 |
| 3 | 0.85 | 171 | -41.0 | 10.1 | 166 |
| 4 | 0.85 | 101 | -21.7 | 10.4 | 93.5 |
| 5 | 0.75 | 53.4 | -10.8 | 10.6 | 44.1 |
| 6 | 0.8 | 30.4 | -5.8 | 10.8 | 20.4 |
| 7 | 0.7 | 28 | -10 | 11.2 | 19.6 |

**Figure S3**. Capacitance measurements in 0.5 M sulfuric acid, with scan rates between 10 and 200 mV/s. Current densities values were taken from the at 0 V vs RHE.

***b) HER activities for NiTiOx***

**
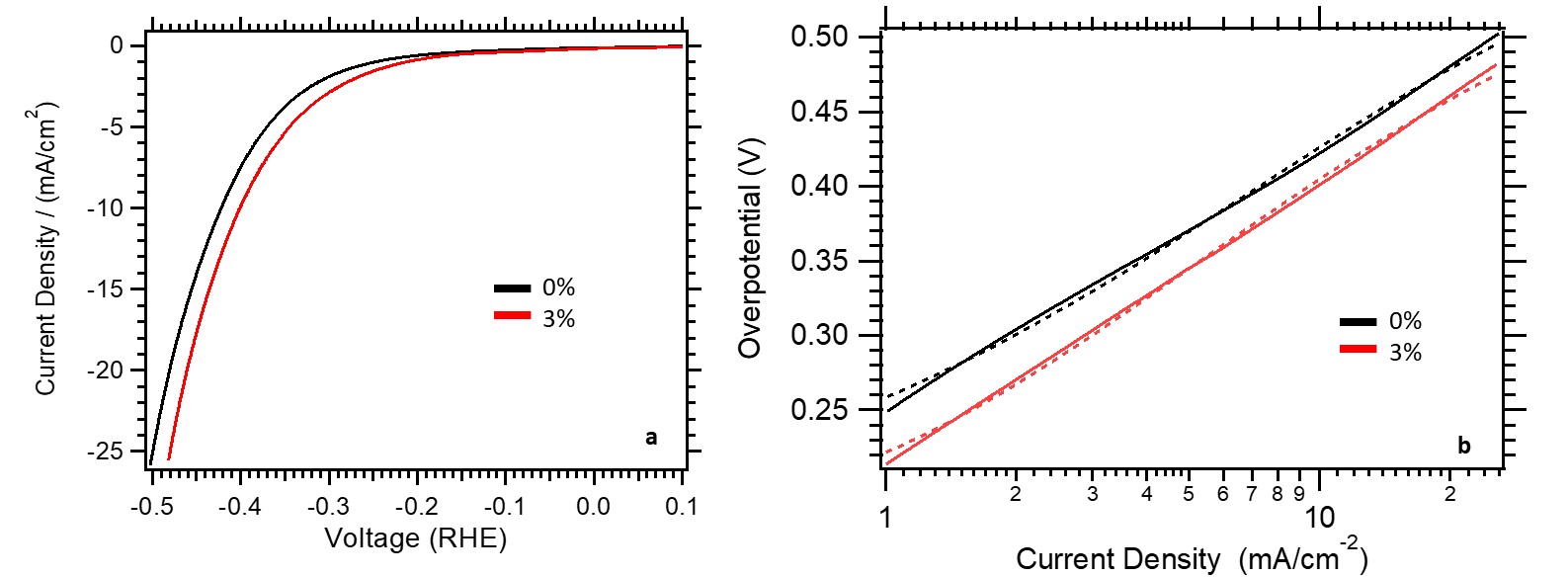
**

**Figure S4.** **a.** LSVs measurements in 0.5 M H_2_SO_4_ for NiTi sample that was not thermally treated (*i.e.*, is covered with native oxide NiTiO_x_) under 0 and 3% strain. **b.** Tafel plots.

**Oxygen Evolution Activities of TiO_2_ Under Tensile Strain**

**Figure S5.** Overpotentials measured at 0.5 mA/cm^2^ and current densities measured at 0.5 V at each strain conditions, in 1M NaOH.


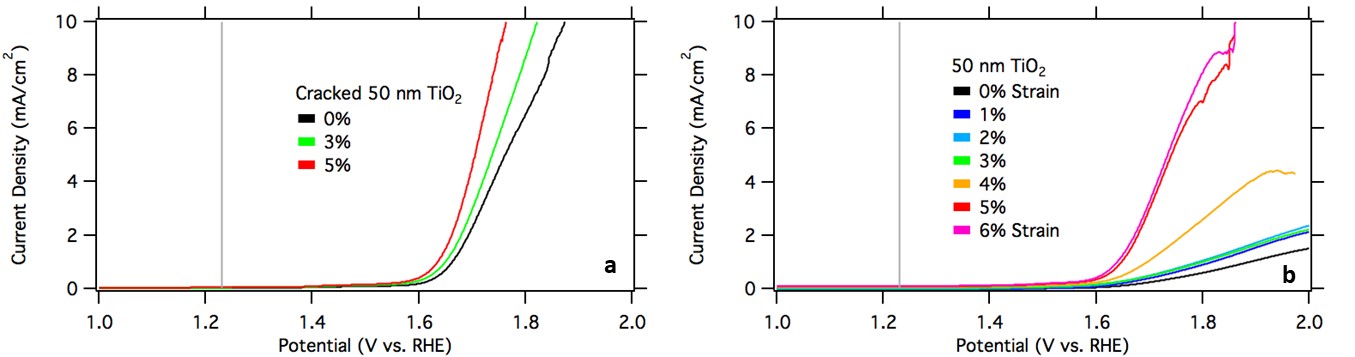


**Figure S6.** **a.** Sample was left at 7% strain overnight to intentionally crack the surface. LSVs in 1 M NaOH were collected for purposely cracked TiO_2_ film at 0, 3 and 5% strain. **b**. LSV for pristine TiO_2_ film was given for comparison. Higher OER activities for purposely cracked sample or sample strained pass 3% are likely due to the opening of surface fissures leading to the exposure of Ni from NiTi substrate, which in its oxidized form (NiO_x_) is more catalytically active than TiO_2_.

**THEORY**

**DFT Calculations**

All plane-wave density functional theory (PW-DFT) calculations were performed using the Vienna Ab-Initio Simulation Package (VASP)[^4^](#_ENREF_4)^,^[^5^](#_ENREF_5) with the most recent projector augmented wave (PAW)[^6^](#_ENREF_6)^,^[^7^](#_ENREF_7) pseudopotentials available. The spin-unrestricted calculations utilized the PBE functional in conjunction with a Hubbard *U*, a Coulombic parameter characterizing repulsion between 3*d* electrons. We implemented a *U* value of 4.2 eV as recommended by Morgan and Watson to recover the presence of localized electrons forming Ti^3+^ states.[^8^](#_ENREF_8) Our experimental lattice constant for the unstrained system was a = 4.59373 Å, c = 2.95812 Å and the unit cell was sliced to expose the (110) facet and grown to a (4 x 2) surface.[^9^](#_ENREF_9) The bottom of our surface was saturated with hydrogens with effective nuclear charges of +4/3 and +2/3 e in order to avoid the spurious effects of slab thickness on surface and adsorption energies as recommended by Kowalski, et. al.[^10^](#_ENREF_10)^,^[^11^](#_ENREF_11) For all calculations presented here, the PAW basis sets were expanded to 520 eV and strict convergence criteria were instituted for electronic (geometric) relaxations to 10^-6^ (10^-5^) eV, with the precision of the calculations set to be accurate. Lattice expansion for strain of 1%, 2%, and 3% was applied uniformly along the axes. The formation energy of an oxygen vacancy was determined by the equation: E_form_ (V_O_)= E_surf+vac_ – E_surf_ + ½μ_O2_, where E_surf+vac_ is the defect surface with an oxygen vacancy, E_surf_ is the total energy of the stoichiometric surface, and μ_O2_ is the total energy of diatomic oxygen. For our adsorption energy, E_H*_ = E_surf+H*_ – E_surf_ - ½E_H2_, where E_surf+H_ is the surface with a single hydrogen adsorbed, E_surf_ is the total energy of the surface, and E_H2_ is the total energy of diatomic hydrogen.

For our free energy:

$${\Delta G}_{H*}=G_{Surf+H*}-G_{1/2H_{2}}-G_{Surf}$$

i.e.

$$G=E+ZPE-T\Delta S$$

where $G_{H*}$ is the free energy of the system with adsorbed hydrogen on the surface and $G_{1/2H_{2}}$ is the halved free energy of diatomic hydrogen. The zero-point energy (ZPE) was calculated from vibrational frequency calculations of diatomic hydrogen and hydrogen adsorbed on the surface (only frequencies of global minima structures were calculated):

$$ZPE=\sum_{i=1}^{n} \frac{1}{2}hv_{i}$$

These frequencies were also evaluated to assess the vibrational entropic contributions to adsorbed hydrogen:

$$S_{vib}=k_{B}\sum_{i=1}^{n} \left[ \frac{hv}{k_{B}T(e^{\frac{hv}{k_{B}T}}-1)}-ln\left( 1-e^{\frac{-hv}{k_{B}T}} \right) \right]$$

For diatomic hydrogen, we utilized NIST tables for the standard entropy.[^12^](#_ENREF_12) For the surface, the free energy was the total energy of the surface as frequency calculations would be prohibitively expensive for the size of this surface.

Due to presence of many local minima for E_surf+H_ (occurs most frequently with adsorption of hydrogen on reduced rutile TiO_2_(110)), we incorporated statistical analysis in order evaluate the Boltzmann probability of the presence of the local minimum. This is performed through use of the Boltzmann probability for *i*-th configuration (*P_i_*) by taking the Boltzmann distribution of each minimum ($e^{-E_{i}/k_{B}T}$) divided by the sum of the distributions of all relevant low energy minima:

$P_{i}=\frac{e^{-E_{i}/k_{B}T}}{\Sigma e^{-E_{i}/k_{B}T}}$,

where *E_i_* is the *i*-th configuration energy of a local minimum for $E_{surf+H}$, $k_{B}$ is the Boltzmann constant, and *T* is the temperature at 22°C or 293.15 K.

As noted in Table S4 below, a considerable distribution occurs amongst the three lowest minima of hydrogen adsorbed on reduced rutile TiO_2_(110). This resulted in us, appropriately, Boltzmann-weighting $E_{surf+H}$ (${\sum P*E}_{surf+H}$) for subsequent free energy calculations in order to represent the ensemble of hydrogen adsorption sites. This effect is negligible for hydrogen adsorption on the stoichiometric surface due to the dominance of the global minimum (Table S5, Figure S8).

We note that for the reduced rutile TiO_2_(110) surface, the two electrons may localize at a number of sites. In other theoretical studies, Calzado, et. al.[^13^](#_ENREF_13) and Zhang, et. al.[^14^](#_ENREF_14) found one electron localizing at a Ti_6f_ near the vacancy and the other at a subsurface Ti; Deskins, et. al[^15^](#_ENREF_15). determined a considerable number of localization combinations all within 0.3-0.4 eV of each other with subsurface Ti sites (in the second layer) being particularly favorable; Kowalski, et. al.[^10^](#_ENREF_10)^,^[^11^](#_ENREF_11) noted in their *ab initio* molecular dynamics study that while the two electrons preferentially populated the subsurface electrons in the second layer, they were able to hop and visit other sites in the surface or third layer of atoms.[^11^](#_ENREF_11)^,^[^13-15^](#_ENREF_13) Similarly, experimental studies also suggest the possibility of different sites captured depending on technique used. In Minato, et. al.’s[^16^](#_ENREF_16) STM measurements, they found localization of electrons at surface Ti_5f_ sites; in contrast, Krüger, et. al.’s[^17^](#_ENREF_17) photoelectron diffraction study identifies localization to distributed over several surface and subsurface Ti sites with the maximum charge on the subsurface Ti site (in the second layer) and a second, smaller charge on surface Ti sites (Ti_6f_ or Ti_5f_).[^16^](#_ENREF_16)^,^[^17^](#_ENREF_17) In our non-strained surface, we had results similar to Kowalski, et. al.[^11^](#_ENREF_11) and Deskins, et. al.[^15^](#_ENREF_15) with the electrons localizing on subsurface Ti atoms in the second layer for all surfaces, with no strain and with strain (see **Figure S7** below). Although we were able to find other minima of the defective surfaces where the two electrons localized at the surface or one electron localized on a surface Ti and one electron remained delocalized, these were significantly higher energy minima of >0.5 eV as compared to where the two electrons localized on subsurface Ti atoms in the second layer of rutile TiO_2_(110).


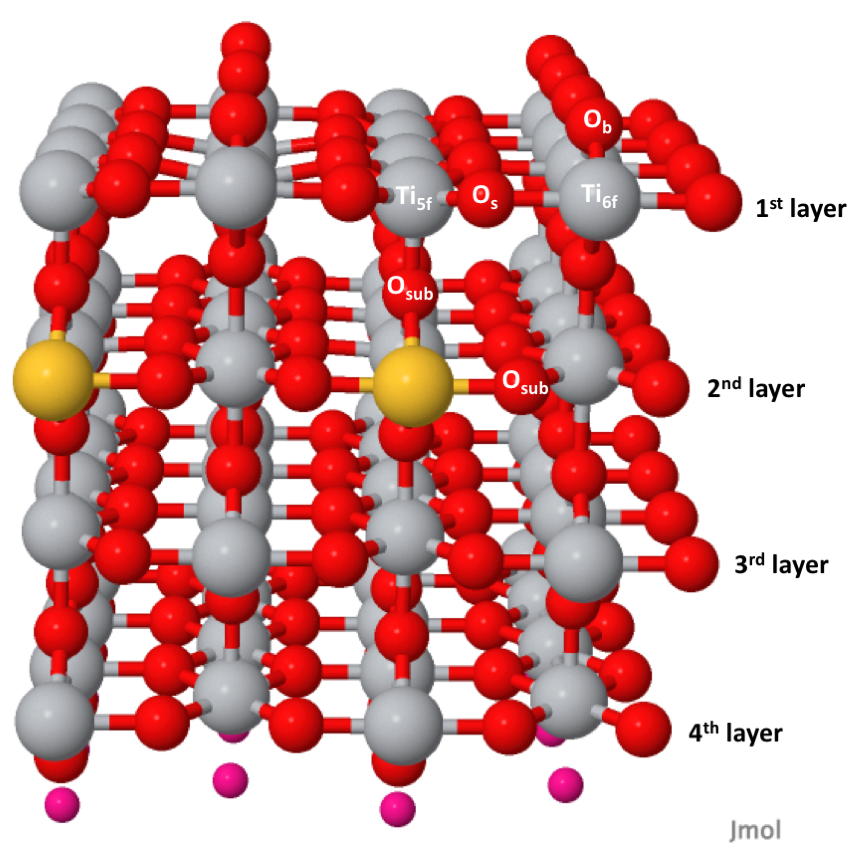


**Figure S7**. Our (4 x 2) surface with (110) facet exposed. Ti atoms in gray, oxygen atoms in red, Ti^3+^ atoms with electrons localized on them in yellow, and 4/3, 1/3 nuclear charged hydrogen atoms in pink. Atoms are designated as follows: Ti_5f_ and Ti_6f_ for the five- or six-fold coordinated Ir atoms; O_b_ for bridging oxygens; O_s_ for in-plane surface oxygen atoms; O_sub_ for sub-surface oxygen atoms. In (110) TiO_2_, Ti_5f_ is coordinated to four O_s_ and one O_sub_ atoms; Ti_6f_ is coordinated to two O_b_, two O_s_, and two O_sub_ atoms.

A previous study with cobalt(II) oxide strained through nanostructuring (*i.e.*, static strain) demonstrates how tensional strain affects H* binding energies (ΔG_H*_)[^18^](#_ENREF_18). In their work, it is shown that an increase in tensional strain from 0 to 4% leads to increase in ΔG_H*_ from negative to positive values, with optimal ΔG_H*_ around 0 eV (*i.e.*, Sabatier principle) achieved at 3% strain. Here, for reduced TiO_2_, the same trend does not occur and corresponds more closely to trends established for metal surfaces, where compression of the crystalline lattice results in weaker adsorption and expansion results in stronger adsorption.[^19^](#_ENREF_19) In general, the adsorption strength of hydrogen is weakened in the presence of an V_O_ (Table S6) with the local minima of hydrogen configurations choosing bridging oxygen sites in the row opposite of the oxygen vacancy. We summarize the adsorption free energies plotted against strain Figure 4a with three of the minima of hydrogen adsorbed on a defective surface in Figure 4b (I-III). More minima are visualized in the Figure S9 (I-IV) as well as their relative energies (Table S7). Moreover, adsorption sites are dependent on strain with the lowest three configurations changing order depending on the strain applied. This suggests that strain may influence the mechanism of water splitting leading to H_2_ evolution in more complex ways than simply changing the adsorption strength of hydrogen: these different local minima may lead to unique reaction pathways depending on the induced strain at the surface. In Yu, et. al.’s work on the oxygen reduction reaction on various metals, they found adsorption strength of oxygen adsorption often corresponded to different rate determining steps in the reaction pathway.[^20^](#_ENREF_20) We note that the formation energy required to create an oxygen vacancy decreases with increasing strain from 2.86 eV (unstrained) to 2.51 eV (strained at 3%) (Table S3). At greater strain, this may lead to an increase in defects in the form of oxygen vacancies, potentially leading to more active sites and subsequently improved HER performance.

**Table S3**. Formation Energy of Oxygen Vacancy in TiO_2_

| Strained TiO_2_ System | E_form_ (eV) |
| --- | --- |
| 0% | 2.86 |
| 1% | 2.78 |
| 2% | 2.66 |
| 3% | 2.51 |

**Table S4**. Global Minima Adsorption Energy (E_H_) of H on Stoichiometric and Strained TiO_2_

|  | 0% | 1% | 2% | 3% |
| --- | --- | --- | --- | --- |
| System | E_H*_ (eV) | E_H*_ (eV) | E_H*_ (eV) | E_H*_ (eV) |
| Stoichiometric | -0.84 | -0.89 | -0.96 | -1.03 |
| Reduced | -0.59 | -0.61 | -0.78 | -0.86 |


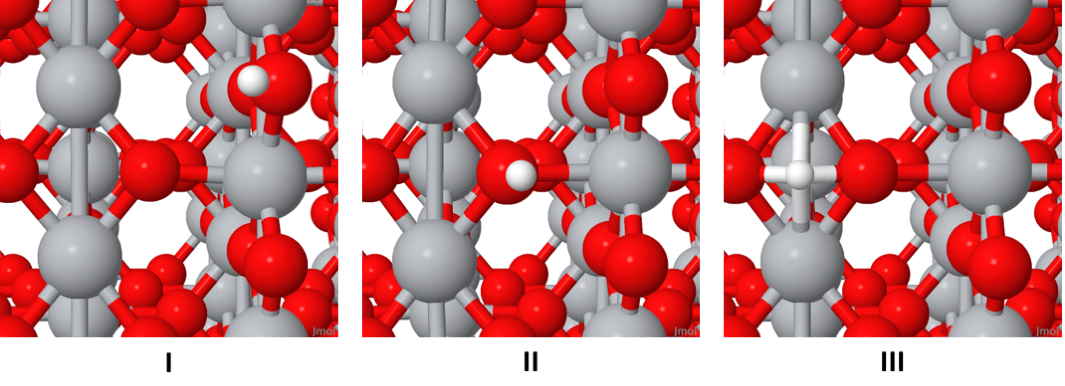


**Figure S8**. Local minima of adsorbed hydrogen on the stoichiometric rutile TiO_2_(110) surface. The same minima were found on the strained surfaces, but with slightly different relative energies detailed below in **Table S5**.

**Table S5**. Relative Adsorption Energy (∆E_H_) * of H on Stoichiometric Strained TiO_2_

|  | 0% | 1% | 2% | 3% |
| --- | --- | --- | --- | --- |
| Isomer | ∆E_H*_ (eV) | ∆E_H*_ (eV) | ∆E_H*_ (eV) | ∆E_H*_ (eV) |
| I | 0.00 | 0.00 | 0.00 | 0.00 |
| II | 1.08 | 1.07 | 0.98 | 0.89 |
| III | 3.09 | 2.46 | 2.38 | 2.29 |

* ∆E_H_ = E_glob_ – E_loc_, the relative adsorption showcases how more unstable a local minimum is compared to the global minimum

**Table S6**. Boltzmann-weighted Adsorption Free Energies (∆G_Boltz,H*_) of H on Stoichiometric and Strained TiO_2_*

|  | 0% | 1% | 2% | 3% |
| --- | --- | --- | --- | --- |
| System | ∆G_Boltz,H*_ (eV) | ∆G_Boltz,H*_ (eV) | ∆G_Boltz,H*_ (eV) | ∆G_Boltz,H*_ (eV) |
| Stoichiometric | -0.69 | -0.75 | -0.80 | -0.87 |
| Reduced | -0.39 | -0.44 | -0.62 | -0.70 |

*Note, in our considerations of G = E – ZPE – TS for free energy of adsorbed local minima, only E is Boltzmann-weighted. The ZPE and vibrational S draw from frequency calculations of the global minimum.


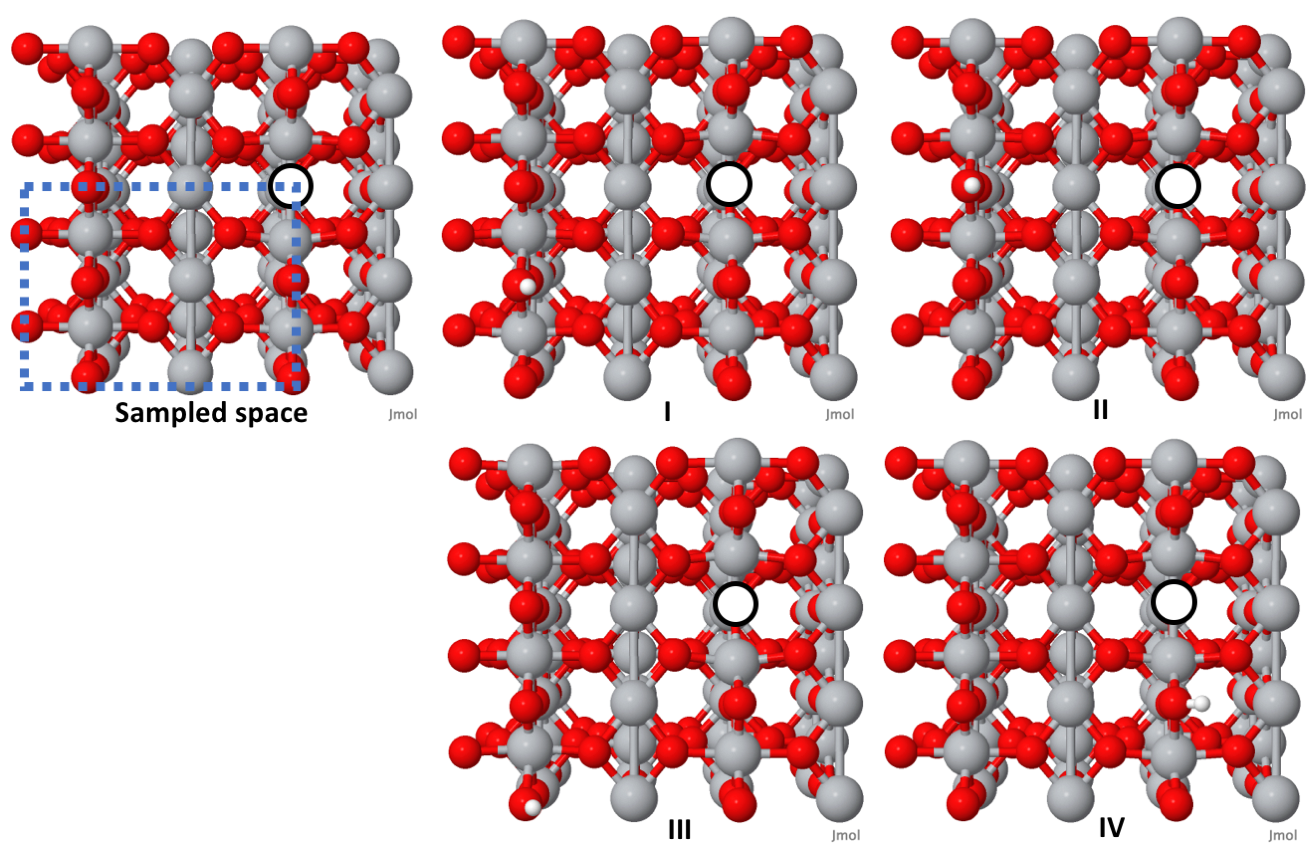


**Figure S9**. Local minima of adsorbed hydrogen on the defective rutile TiO_2_(110) surface. The same minima were found on the strained surfaces, but in a different order with corresponding changes to the relative energies detailed below. For our labels of isomers I-IV, we utilize the local minima of the unstrained surface and then report changes in our table below, **Table S7**.

**Table S7**. Relative Adsorption Energy (∆E_H_) * of H on Reduced Strained TiO_2_

| 0% | | | 1% | | | 2% | | | 3% | | |
| --- | --- | --- | --- | --- | --- | --- | --- | --- | --- | --- | --- |
| Iso. | P_295K_ | ∆E_H*_ (eV) | Iso. | P_295K_ | ∆E_H*_ (eV) | Iso. | P_295K_ | ∆E_H*_ (eV) | Iso. | P_295K_ | ∆E_H*_ (eV) |
| I | 59.06% | 0.00 | I | 50.15% | 0.00 | III | 78.36% | 0.00 | I | 53.95% | 0.00 |
| II | 23.31% | 0.01 | III | 28.40% | 0.00 | II | 21.47% | 0.02 | III | 30.10% | 0.00 |
| III | 17.63% | 0.02 | II | 21.06% | 0.01 | I | <0.01% | 0.15 | II | 15.95% | 0.02 |
| IV | <0.01% | 0.32 | IV | <0.01% | 0.33 | IV | <0.01% | 0.49 | IV | <0.01% | 0.54 |

* ∆E_H_ = E_glob_ – E_loc_, the relative adsorption showcases how more unstable a local minimum is compared to the global minimum


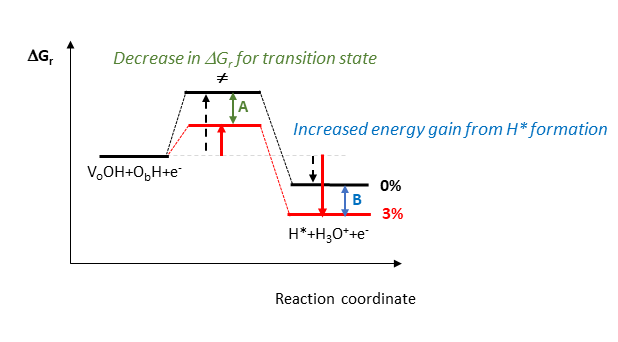


**Figure S10.** Putative reaction pathway that shows different scenarios where stronger H* adsorption could improve HER activities: **A.** by lowering energy of the transition state (≠)**, B.** by increasing the energy gain from H* formation. Here, first Volmer step is broken down to H_2_O adsorption to Vo, dissociation to OH* and O_b_H* and reduction.

(*) symbolizes a surface bound state. Note that strain also impacts energies of other intermediates and transition states, which was not studied in this work. To determine the full reaction coordinate as a function of strain we would need calculations for each step in the reaction pathway, including adsorbed water and OH as well as the reaction barrier to water splitting.

**References**

1 Seh, Z. W. *et al.* Combining theory and experiment in electrocatalysis: Insights into materials design. *Science* **355**, eaad4998, doi:10.1126/science.aad4998 (2017).

2 Roger, I., Shipman, M. A. & Symes, M. D. Earth-abundant catalysts for electrochemical and photoelectrochemical water splitting. *Nature Reviews Chemistry* **1**, 0003, doi:10.1038/s41570-016-0003 (2017).

3 Benson, E. E. *et al.* Semiconductor-to-Metal Transition in Rutile TiO2 Induced by Tensile Strain. *Chemistry of Materials* **29**, 2173-2179, doi:10.1021/acs.chemmater.6b04881 (2017).

4 Kresse, G. & Hafner, J. Ab initio molecular-dynamics simulation of the liquid-metal–amorphous-semiconductor transition in germanium. *Physical Review B* **49**, 14251 (1994).

5 Kresse, G. & Furthmüller, J. Efficiency of ab-initio total energy calculations for metals and semiconductors using a plane-wave basis set. *Computational materials science* **6**, 15-50 (1996).

6 Kresse, G. & Joubert, D. From ultrasoft pseudopotentials to the projector augmented-wave method. *Physical Review B* **59**, 1758 (1999).

7 Blöchl, P. E. Projector augmented-wave method. *Physical review B* **50**, 17953 (1994).

8 Morgan, B. J. & Watson, G. W. A density functional theory+ U study of oxygen vacancy formation at the (110),(100),(101), and (001) surfaces of rutile TiO2. *The Journal of Physical Chemistry C* **113**, 7322-7328 (2009).

9 Wyckoff, R. W. G. *Crystal Structures*. (Wiley, 1963).

10 Kowalski, P. M., Meyer, B. & Marx, D. Composition, structure, and stability of the rutile TiO 2 (110) surface: Oxygen depletion, hydroxylation, hydrogen migration, and water adsorption. *Physical Review B* **79**, 115410 (2009).

11 Kowalski, P. M., Camellone, M. F., Nair, N. N., Meyer, B. & Marx, D. Charge localization dynamics induced by oxygen vacancies on the TiO 2 (110) surface. *Physical review letters* **105**, 146405 (2010).

12 Chase, M., Davies, C., Downey, J. & Frurip, D. J. Phys. Chem. Ref. Data. *JANAF Thermochemical Tables* **4** (1998).

13 Calzado, C. J., Hernández, N. C. & Sanz, J. F. Effect of on-site Coulomb repulsion term U on the band-gap states of the reduced rutile (110) Ti O 2 surface. *Physical Review B* **77**, 045118 (2008).

14 Zhang, H., Soon, A., Delley, B. & Stampfl, C. Stability, structure, and electronic properties of chemisorbed oxygen and thin surface oxides on Ir (111). *Physical Review B* **78**, 045436 (2008).

15 Deskins, N. A., Rousseau, R. & Dupuis, M. Distribution of Ti3+ surface sites in reduced TiO2. *The Journal of Physical Chemistry C* **115**, 7562-7572 (2011).

16 Minato, T. *et al.* The electronic structure of oxygen atom vacancy and hydroxyl impurity defects on titanium dioxide (110) surface. *The Journal of chemical physics* **130**, 124502 (2009).

17 Krüger, P. *et al.* Defect states at the TiO 2 (110) surface probed by resonant photoelectron diffraction. *Physical review letters* **100**, 055501 (2008).

18 Ling, T. *et al.* Activating cobalt(II) oxide nanorods for efficient electrocatalysis by strain engineering. *Nature Communications* **8**, 1509, doi:10.1038/s41467-017-01872-y (2017).

19 Mavrikakis, M., Hammer, B. & Nørskov, J. K. Effect of Strain on the Reactivity of Metal Surfaces. *Physical Review Letters* **81**, 2819-2822 (1998).

20 Yu, T. H. *et al.* Finding correlations of the oxygen reduction reaction activity of transition metal catalysts with parameters obtained from quantum mechanics. *The Journal of Physical Chemistry C* **117**, 26598-26607 (2013).
